# HOIMamba: Bidirectional State-Space Modeling for Monocular 3D Human–Object Interaction Reconstruction

**DOI:** 10.3390/biomimetics11030214

**Published:** 2026-03-17

**Authors:** Jinsong Zhang, Yuqin Lin

**Affiliations:** School of Computer Science, Big Data and Software, Fuzhou University, Fuzhou 350108, China; jinszhang@fzu.edu.cn

**Keywords:** human–object interaction, monocular 3D reconstruction, state-space model, Mamba, contact-aware reconstruction

## Abstract

Monocular 3D human–object interaction (HOI) reconstruction requires jointly recovering articulated human geometry, object pose, and physically plausible contact from a single RGB image. While recent token-based methods commonly employ dense self-attention to capture global dependencies, isotropic all-to-all mixing tends to entangle spatial-geometric cues (e.g., contact locality) with channel-wise semantic cues (e.g., action/affordance), and provides limited control for representing directional and asymmetric physical influence between humans and objects. This paper presents HOIMamba, a state-space sequence modeling framework that reformulates HOI reconstruction as bidirectional, multi-scale interaction state inference. Instead of relying on symmetric correlation aggregation, HOIMamba uses structured state evolution to propagate interaction evidence. We introduce a multi-scale state-space module (MSSM) to capture interaction dependencies spanning local contact details and global body–object coordination. Building on MSSM, we propose a spatial-channel grouped SSM (SCSSM) block that factorizes interaction modeling into a spatial pathway for geometric/contact dependencies and a channel pathway for semantic/functional correlations, followed by gated fusion. HOIMamba further performs explicit bidirectional propagation between human and object states to better reflect asymmetric reciprocity in physical interactions. We evaluate HOIMamba on two public benchmarks, BEHAVE and InterCap, using Chamfer distance for human/object meshes and contact precision/recall induced by reconstructed geometry. HOIMamba achieves consistent improvements over representative prior methods. On the BEHAVE dataset, it reduces human Chamfer distance by 8.6% and improves contact recall by 13.5% compared to the strongest Transformer-based baseline, with similar gains observed on the InterCap dataset. Ablation studies on BEHAVE verify the contributions of state-space modeling, multi-scale inference, spatial-channel factorization, and bidirectional interaction reasoning.

## 1. Introduction

Recovering detailed 3D human–object interactions (HOIs) from a single RGB image is a fundamental problem in computer vision, with broad applications in augmented reality [1,2,3], human–robot interaction [4,5,6,7], and digital content creation [8,9]. Unlike independent human or object reconstruction, HOI reconstruction requires jointly inferring articulated human pose, object geometry, and their physically plausible interaction states. This task is particularly challenging due to severe depth ambiguity, frequent mutual occlusion, and the asymmetric nature of physical interactions, all of which must be inferred from a single 2D observation. In many embodied settings, the goal is not only to recover shapes, but also to infer interaction states that are useful for downstream manipulation and contact reasoning. Moreover, in collaborative robotics, recovering contact-relevant interaction geometry helps a robot reason about grasp/support regions and safe proximity during co-manipulation.

Early approaches primarily relied on volumetric or implicit representations [10,11] to model occupancy and contact consistency between humans and objects. While effective in capturing interaction realism, these methods are computationally expensive and often lack explicit and controllable mechanisms for structured interaction reasoning across entities. More recently, token-based frameworks [12,13] have become the dominant paradigm. By representing humans and objects as discrete tokens and leveraging Transformer [14] architectures, these methods model cross-entity correlations through global self-attention and achieve strong overall reconstruction performance.

Standard attention is flexible, but its inductive bias does not match fine-grained HOI understanding. Self-attention mixes token features in an isotropic, all-to-all way. This mixing often entangles spatial-geometric relations with channel-wise semantic cues. For example, the model may mix “where contact happens” with “what the action means.” This entanglement helps global context aggregation. However, it makes local interaction reasoning harder, leading to inaccurate estimation. Moreover, the model also struggles with asymmetric reciprocity, which is common in physical interaction. As a result, many methods predict interaction states that look globally consistent, while these predictions are often locally inaccurate in contact-sensitive regions.

HOI reconstruction is not only a correlation modeling problem. It is also a reciprocal state inference problem under physical constraints, and these constraints are often asymmetric. For example, a hand configuration strongly constrains a grasped object pose. In contrast, object geometry provides weaker feedback to the global human pose. This directional dependency is common in interaction perception, and it resembles how biological systems accumulate evidence through structured updates. Therefore, a suitable architecture should model these properties, rather than only capturing global correlations.

Structured State-Space Models (SSMs) [15,16] are a recent alternative to Transformers. Specifically, Mamba [16] replaces quadratic attention with selective state evolution in linear time, which not only improves scalability, but also enables controlled information propagation through latent states. This property provides a useful prior for directional and structured dependencies. In HOI reconstruction, a latent state can represent an interaction condition, which encodes contact configuration and spatial constraints between entities. This alignment is conceptually appealing for HOIs. However, state-space modeling for complex 3D interaction reasoning remains underexplored.

**Figure 1:**
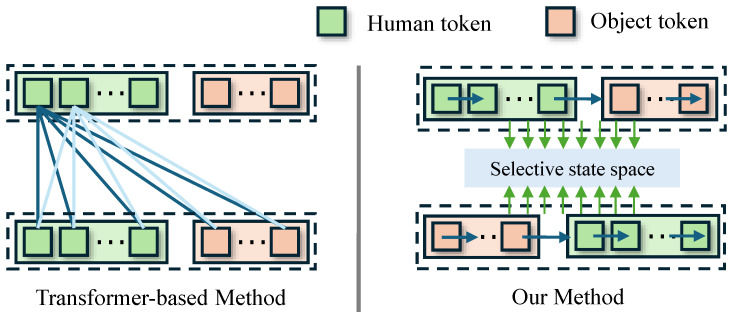
Comparison with previous methods. Prior approaches [12,13] use dense all-to-all attention. This design couples spatial and semantic cues through symmetric feature mixing. In contrast, the proposed method factorizes interaction reasoning into structured state updates. This design supports disentangled modeling of geometric and semantic dependencies through state-space evolution.

In this work, we introduce HOIMamba, a novel state-space framework for monocular 3D human–object interaction reconstruction. As illustrated in Figure 1, HOI reconstruction is reformulated as a problem of bidirectional, multi-scale interaction state inference, where human and object states are iteratively co-estimated rather than inferred through a single static correlation step.

To this end, we design a spatial-channel grouped state-space modeling (SCSSM) block, built upon a multi-scale state-space module (MSSM). The proposed design explicitly decomposes interaction reasoning into two complementary pathways: (1) a spatial state pathway that processes token sequences to capture long-range geometric dependencies and fine-grained contact relationships across multiple spatial scales with linear complexity; and (2) a channel state pathway that operates across feature channels to model semantic and functional correlations. The two pathways are fused through a gated mechanism, enabling structured and efficient reasoning about both where interaction occurs and how it is semantically realized.

Furthermore, HOIMamba incorporates an explicit bidirectional reasoning scheme, in which information propagates separately from human to object and from object to human. This design matches the asymmetric nature of physical interactions, and lets the model capture directional influence patterns, which are hard to represent with symmetric attention-based formulations.

We evaluate HOIMamba on two standard benchmarks, BEHAVE [17] and InterCap [18]. Experimental results demonstrate that the proposed method consistently outperforms prior Transformer-based approaches [12]. The gains are especially strong on contact-aware evaluation metrics. These results demonstrate that state-space modeling, when equipped with appropriate structural inductive biases, provides an effective and scalable paradigm for disentangled HOI understanding.

Our main contributions are summarized as follows:

- We propose HOIMamba, a bidirectional state-space framework for monocular 3D human–object interaction reconstruction.
- We introduce a multi-scale state-space module that captures hierarchical interaction structures and fine-grained contact details.
- We design a spatial-channel grouped SSM block that decouples geometric and semantic interaction reasoning into parallel, efficient state-space pathways.
- We achieve state-of-the-art performance on two benchmarks, with substantial gains on contact-sensitive evaluation metrics.

The remainder of this paper is organized as follows. Section 2 reviews related work. Section 3 details the proposed state-space interaction modeling framework. Experimental results are presented in Section 4, followed by concluding remarks in Section 6.

## 2. Related Work

### 2.1. 3D Human Reconstruction

Reconstructing 3D human geometry and pose from monocular images has been extensively studied in computer vision. Early approaches [19,20,21] focused on parametric body models such as SMPL [22], estimating body pose and shape through optimization-based fitting pipelines. While effective under controlled settings, these methods often suffer from local minima and limited robustness in the presence of occlusion or complex poses.

Learning-based approaches [23,24,25,26] subsequently became dominant, leveraging convolutional or Transformer-based architectures to regress human pose and shape directly from images. Recent methods further improve reconstruction quality by incorporating implicit representations, volumetric features, or surface-based modeling, enabling more detailed geometry recovery. Despite significant progress, most existing human reconstruction methods assume the human body to be an isolated entity and do not explicitly consider interaction with surrounding objects.

These assumptions often fail in HOI scenarios. Objects can occlude the human body, and contact can impose strong pose constraints. Additionally, interactions can also introduce task-specific deformations. These factors create ambiguities. Therefore, a human-only reconstruction model cannot resolve these ambiguities reliably.

In this paper, we propose HOIMamba, which jointly models human and object states through multi-scale and double-path structured state-space evolution.

### 2.2. 3D Human–Object Interaction Reconstruction

Reconstructing 3D human–object interactions (HOI) from visual observations has attracted growing interest. This problem matters for augmented reality, embodied AI, and human–robot interaction. Early works [10,11] often estimated the human body and the object geometry separately. These methods then applied post hoc refinement to reduce interpenetration. They also used refinement to enforce coarse contact constraints. This pipeline is conceptually simple. However, it does not perform explicit interaction reasoning, leading to failure under severe occlusion. Additionally, this pipeline also fails in close-contact scenarios.

Large-scale datasets such as BEHAVE [17] and InterCap [18] have changed the landscape. These datasets enable end-to-end joint reconstruction frameworks. Such frameworks aim to infer human pose, object geometry, and interaction states at the same time. CONTHO [12] is a representative approach. It explicitly enforces contact consistency to improve interaction fidelity.

However, existing HOI reconstruction methods still have inherent limitations. Dense all-to-all attention has quadratic computational cost, which mixes spatial-geometric relations with semantic correlations. This mixing not only makes localized contact reasoning harder, but it also makes physical-constraint reasoning more difficult. Graph-based formulations [13] add topology priors to the interaction inference stage. However, these kind of methods require carefully designed adjacency structures. In addition, both paradigms struggle with asymmetric physical interactions. In HOIs, influence between humans and objects is bidirectional, which is also uneven and directional.

These observations motivate alternative modeling paradigms that should go beyond symmetric correlation and support structured and directional interaction reasoning. In this paper, we propose a novel formulation that takes the HOI reconstruction as a process of bidirectional, multi-scale interaction state inference using structured state-space models. Under this formulation, the proposed HOIMamba outperforms prior methods on the quality of reconstruction results.

### 2.3. State-Space Models

State-space models (SSMs) [15,27,28] have recently emerged as an effective alternative to attention-based architectures for modeling long-range dependencies. SSMs parameterize feature evolution through selective state updates. This design not only achieves linear computational complexity, but it also preserves strong expressive capacity. Specifically, Mamba [16] is a representative SSM architecture. It introduces selective scanning and parallelizable recurrence, enabling efficient modeling of long sequences. Moreover, it also provides controlled information flow, which is useful for structured reasoning. Additionally, SSMs have shown strong results in language modeling [29,30] and vision tasks [31,32,33]. These gains are notable in settings that require structured dependency modeling. Additionally, there are many recent works that apply SSMs to 3D object detection [34,35], 3D medical image segmentation [36,37,38], and 3D motion generation [39,40]. These works demonstrate the effectiveness of state-space modeling for structured spatial and temporal reasoning in diverse 3D understanding and generation scenarios. However, most existing Mamba-based works focus on single-stream modeling of either spatial or temporal signals. The application of structured state-space modeling to reciprocal human–object interaction reasoning remains largely unexplored. In the context of HOI reconstruction, state-space modeling offers a natural mechanism to represent latent interaction states, such as contact configuration and spatial constraints, and to propagate these states in a directional and controllable manner.

Motivated by these properties, our work leverages bidirectional and multi-scale state-space modeling to capture asymmetric human–object interactions. By reformulating HOI reconstruction as a process of structured state inference rather than symmetric feature correlation, we provide a complementary and scalable alternative to existing Transformer-based approaches.

## 3. Method

**Figure 2:**
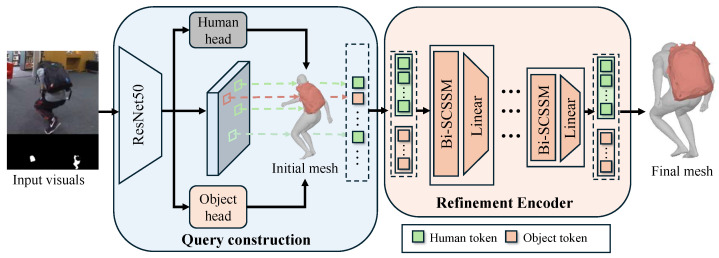
Overview of the HOIMamba pipeline. The query construction stage produces coarse human and object states, which are then refined through a bidirectional state-space refinement encoder that models structured interaction dependencies.

Given a single RGB image depicting a human–object interaction (HOI) and a 3D template mesh of the target object, the goal is to reconstruct the 3D human mesh and estimate the 6D pose of the object. We propose HOIMamba, an end-to-end framework that reformulates HOI reconstruction as a process of bidirectional, multi-scale interaction state inference using structured state-space models (SSMs). As illustrated in Figure 2, HOIMamba first estimates coarse human and object states to provide reliable geometric priors, and subsequently refines these states through a bidirectional state-space encoder that explicitly models reciprocal human–object influence. The overall pipeline consists of two stages: an initialization stage and a bidirectional state-space refinement stage.

### 3.1. Initialization

The initialization stage aims to provide strong and reliable geometric priors rather than explicitly modeling interaction. Following prior work [12,13], the input consists of an RGB image $I\in R^{3\times H\times W}$ together with segmentation masks for the human ($S_{h}$) and the object ($S_{o}$). A ResNet-50 backbone extracts a hierarchical feature map $F\in R^{2048\times \frac{H}{32}\times \frac{W}{32}}$.

**Human Initialization.** Following [26], the backbone regresses the parameters of the SMPL-H model [22], including body shape $\beta \in R^{10}$, body pose $\theta_{\mathrm{body}}\in R^{66}$, and hand pose $\theta_{\mathrm{hand}}\in R^{90}$. The SMPL-H function $M_{\mathrm{SMPLH}}\left(\beta ,\theta_{\mathrm{body}},\theta_{\mathrm{hand}}\right)$ produces an initial human mesh $M_{h}^{\mathrm{init}}\in R^{6890\times 3}$ and 3D joints $J^{\mathrm{init}}\in R^{73\times 3}$. For computational efficiency in subsequent refinement, the mesh is downsampled to $M_{h}^{\mathrm{init}}\in R^{431\times 3}$ using a predefined downsampling matrix $D\in R^{431\times 6890}$.
**Object Initialization.** The initial 6D pose of the object is predicted by a lightweight regression head attached to the backbone, yielding rotation $R_{\mathrm{init}}\in SO\left(3\right)$ and translation $T_{\mathrm{init}}\in R^{3}$. Applying the predicted pose to the object template vertices $V_{\mathrm{obj}}^{\mathrm{temp}}\in R^{N_{o}\times 3}$ yields
  (1) $$M_{o}^{\mathrm{init}}=V_{\mathrm{obj}}^{\mathrm{temp}}R_{\mathrm{init}}^{⊤}+T_{\mathrm{init}},$$
  where $N_{o}=64$ denotes the number of sampled object vertices.
**3D-Aware Query Construction.** To integrate visual appearance with 3D geometry, we construct hybrid query tokens. A weak-perspective camera Π is estimated from F to project 3D points onto the image plane. For each 3D point $p\in \{J^{\mathrm{init}},M_{h}^{\mathrm{init}},M_{o}^{\mathrm{init}}\}$, its 2D projection u=Π(p) is computed, and an appearance feature $f_{\mathrm{app}}\in R^{2048}$ is extracted via bilinear grid sampling on F. The appearance feature is concatenated with the corresponding 3D coordinates to form a position-aware query:
  (2) $$\begin{array}{cc} Q_{j} & =[\mathrm{GS}\left(J^{\mathrm{init}},F\right);J^{\mathrm{init}}], \end{array}$$
  (3) $$\begin{array}{cc} Q_{h} & =[\mathrm{GS}\left(M_{h}^{\mathrm{init}},F\right);M_{h}^{\mathrm{init}}], \end{array}$$
  (4) $$\begin{array}{cc} Q_{o} & =[\mathrm{GS}\left(M_{o}^{\mathrm{init}},F\right);M_{o}^{\mathrm{init}}]. \end{array}$$
  The final token sequence is $Q=\left[Q_{j};Q_{h};Q_{o}\right]\in R^{L\times d_{\mathrm{in}}}$, with L=568 and $d_{\mathrm{in}}=2051$.

The segmentation masks are crucial for two reasons: (1) they guide the ResNet-50 backbone to extract interaction-specific features by focusing computation on the relevant image regions, and (2) more importantly, they are used to construct the 3D-aware queries. By projecting the initial 3D human joints/vertices and object vertices onto the image plane using the estimated camera, and then sampling appearance features from F only within the masked regions (via grid sampling), we effectively fuse 2D semantic information with 3D geometry. This grounds the 3D tokens in the 2D image evidence, providing a robust foundation for the subsequent refinement stage.

**Sequence Ordering.** Tokens are concatenated in a fixed semantic order: joints, human vertices, and object vertices. Within each entity, tokens follow predefined joint hierarchies or mesh topologies. The fixed semantic order (joints → human vertices → object vertices, with internal topologies) is designed to impose a structured traversal over the interaction graph. This deterministic ordering allows the state-space models (SSMs) to accumulate interaction evidence in a controlled and spatially meaningful manner, which mimics a progressive scan of the human–object constellation. This design provides a strong structural prior for the SSM, which is crucial for capturing the long-range dependencies between distant body joints and the object, as well as local contacts.

### 3.2. Bidirectional State-Space Refinement Encoder

The core of HOIMamba is a multi-layer refinement encoder built upon structured state-space models (SSMs), which replace quadratic self-attention with linear-complexity state evolution. This design enables efficient and structured modeling of long-range, asymmetric human–object interactions.

#### 3.2.1. Preliminary: State-Space Models

Structured state-space models, such as Mamba [16], model a 1-D input sequence through a latent state that evolves according to

(5) $$\begin{array}{cc} h^{\prime }\left(t\right) & =Ah(t)+Bx(t),\\ y(t) & =Ch(t). \end{array}$$

In practice, we use a discretized selective SSM implementation following [16]. Unlike self-attention, an SSM propagates information through controlled state evolution. This mechanism supports directional and progressive aggregation of interaction cues. Additionally, the recurrent update with input-dependent matrices enables dynamic control over information flow along the sequence. This contrasts with the static, symmetric affinity matrix in attention, and provides a structural bias that aligns with the asymmetric nature of human–object interactions.

#### 3.2.2. Multi-Scale State-Space (MSSM) Block

**Figure 3:**
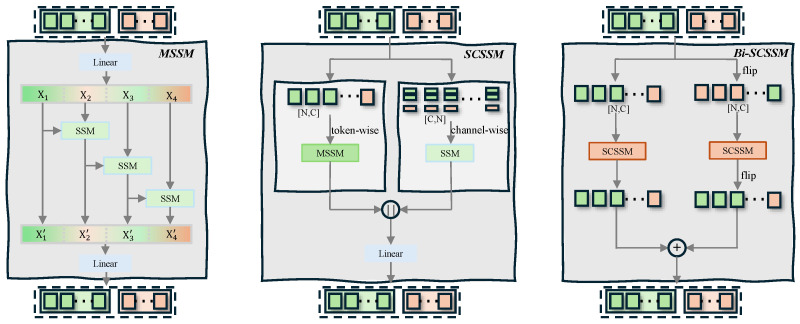
Architecture of the proposed multi-scale state-space module (MSSM) and spatial-channel grouped SSM (SCSSM) block. MSSM captures interaction dependencies at multiple spatial scales through parallel SSM layers with increasing convolutional kernel sizes. SCSSM decouples spatial and channel-wise interaction reasoning into separate pathways, and then applies gated fusion.

The model needs interaction cues at different granularities. Therefore, as shown in Figure 3, we introduce a multi-scale state-space (MSSM) block. The block takes an input $X\in R^{L\times d}$. We first apply LayerNorm to X. We then apply a linear projection to transform the input to a higher-dimensional space.

We split the projected features into G=4 groups along the channel dimension. Denote the groups as ${\left\{X_{g}\right\}}_{g=1}^{G}$. Each $X_{g}$ has shape $R^{L\times (d/G)}$. After that, each group goes through an independent SSM layer, which uses an increasing convolutional kernel size. We set the kernel size as $d_{\mathrm{conv}}^{g}=4g$. The kernel size is designed to increase proportionally with the group index *g*. This design allows the earlier groups (with smaller kernels) to focus on fine-grained, local interaction details (e.g., hand–object contact), while later groups (with larger kernels) capture broader, global spatial dependencies (e.g., whole-body posture relative to the object). This linear scaling provides a simple yet effective way to achieve a spectrum of receptive fields without introducing additional hyperparameters.

The block uses residual information flow across groups. We define the cross-group recurrence as

(6) $$H_{g}={\mathrm{SSM}}_{g}\left(X_{g}+W_{\mathrm{res}}H_{g-1}\right),\;g=1,\ldots ,G,$$

where $H_{0}$ is a learnable projection of $X_{1}$. We concatenate the group outputs, and then project the concatenated features back to the model dimension:

(7) $$\mathrm{MSSM}\left(X\right)=W_{\mathrm{out}}\left[H_{1};H_{2};H_{3};H_{4}\right].$$

This multi-scale design improves contact-sensitive reconstruction accuracy.

#### 3.2.3. Spatial-Channel Grouped SSM (SCSSM) Block

HOI reasoning needs two types of cues. The model needs to know where interaction occurs, which relates to geometry and contact. The model also needs to know how interaction is realized, which relates to affordance and action. To this end, we propose a spatial-channel grouped SSM (SCSSM) block. Unlike previous works, this block explicitly separates spatial reasoning from channel-wise reasoning.

**Spatial State Pathway.** Built on MSSM, this pathway operates along the token dimension, which captures geometric proximity and long-range spatial dependencies:
  (8) $$H_{\mathrm{spa}}=\mathrm{MSSM}\left(X\right).$$
**Channel State Pathway.** This pathway operates along the feature dimension and models semantic correlations across channels. We transpose the input as $X^{⊤}\in R^{d\times L}$. We then apply a Mamba layer to capture channel-wise dependencies:
  (9) $$H_{\mathrm{cha}}^{⊤}=M\left(X^{⊤}\right),$$
  followed by transposition back to $H_{\mathrm{cha}}\in R^{L\times d}$. Compared to channel-wise MLPs or attention, channel SSMs enable progressive and directional feature aggregation, yielding more stable semantic alignment. A channel attention gate is applied:
  (10) $$A_{\mathrm{cha}}=\sigma \left(\mathrm{MLP}(\mathrm{AvgPool}(X))\right),\;{\tilde{H}}_{\mathrm{cha}}=A_{\mathrm{cha}}⊙H_{\mathrm{cha}}.$$
**Fusion.** The final SCSSM output is obtained by fusing both pathways:
  (11) $$\mathrm{SCSSM}\left(X\right)=\mathrm{LayerNorm}\left(W_{f}\left[H_{\mathrm{spa}};{\tilde{H}}_{\mathrm{cha}}\right]+X\right),$$
  where $W_{f}\in R^{d\times 2d}$. The forward pass (SCSSM(X)) processes the sequence in the order of [joints, human vertices, object vertices], which can be interpreted as information flowing from the human to the object (i.e., how the human’s state influences the object’s pose). Conversely, the backward pass processes the reversed order [object vertices, human vertices, joints], simulating information flow from the object back to the human (i.e., how the object’s geometry and pose constrain the human’s posture). By fusing both directions, the model captures the full bidirectional and asymmetric nature of the interaction, leading to more physically plausible reconstructions.

#### 3.2.4. Bidirectional SSM Encoder Layer

To capture asymmetric reciprocal influence, each encoder layer processes the sequence in both forward and reverse orders:

(12) $$\begin{array}{cc} H_{\mathrm{fwd}} & =\mathrm{SCSSM}(X), \end{array}$$

(13) $$\begin{array}{cc} H_{\mathrm{bwd}} & =\mathrm{SCSSM}(\mathrm{Flip}(X)). \end{array}$$

The reversed output is aligned back and fused:

(14) $$H=\mathrm{LayerNorm}\left(H_{\mathrm{fwd}}+\mathrm{Flip}\left(H_{\mathrm{bwd}}\right)\right).$$

Forward and backward propagation correspond to aggregating interaction evidence under different traversal orders of the interaction graph, improving robustness to ordering bias.

#### 3.2.5. Multi-Stage Encoder Architecture

The refinement encoder stacks three bidirectional SSM layers with decreasing hidden dimensions (1024→512→256). A final linear layer predicts 3D coordinate offsets, which are added to the initial coordinates. The refined human mesh is upsampled to full SMPL-H resolution using a predefined upsampling matrix [25]. The final object pose is recovered via Procrustes alignment.

### 3.3. Training Objectives

We train the network end-to-end with a weighted composite objective:

(15) $$L=L_{\mathrm{human}}+L_{\mathrm{object}}+L_{\mathrm{hbox}}.$$

**Human Mesh Loss.** The human loss combines multi-scale vertex supervision, joint supervision, edge-length regularization, and SMPL-H parameter supervision:
  (16) $$L_{\mathrm{human}}=\lambda_{\mathrm{msv}}L_{\mathrm{ms}\text{-}\mathrm{vertex}}+\lambda_{\mathrm{edge}}L_{\mathrm{edge}}+\lambda_{\mathrm{param}}L_{\mathrm{param}}.$$
**Multi-scale vertex loss.** Let $V_{h}^{\left(0\right)}\in R^{N_{0}\times 3}$ denote the predicted coarse human vertices (e.g., $N_{0}=431$), and $V_{h}^{\left(1\right)}$, $V_{h}^{\left(2\right)}$ be the upsampled vertices at two finer resolutions (e.g., $N_{1}=1723$, $N_{2}=6890$). Let the corresponding ground-truth vertices be $V_{h,*}^{\left(s\right)}$ at each scale s∈{0,1,2}. We define
  (17) $$L_{\mathrm{ms}\text{-}\mathrm{vertex}}=\sum_{s\in \{0,1,2\}}\frac{1}{N_{s}}{∥V_{h}^{\left(s\right)}-V_{h,*}^{\left(s\right)}∥}_{1}.$$
  This constrains human reconstruction quality progressively from coarse geometry to full-resolution details.
**Edge-length consistency.** Let E be the edge set of the human mesh topology (at full resolution), and (i,j)∈E indicates an edge between vertices *i* and *j*. We penalize the discrepancy between predicted and GT edge lengths:
  (18) $$L_{\mathrm{edge}}=\frac{1}{\left|E\right|}\sum_{(i,j)\in E}\left|{∥V_{h,i}^{\left(2\right)}-V_{h,j}^{\left(2\right)}∥}_{2}-{∥V_{h,*,i}^{\left(2\right)}-V_{h,*,j}^{\left(2\right)}∥}_{2}\right|.$$
**SMPL-H parameter loss.** Let θ denote the predicted SMPL-H parameters (e.g., body pose, hand pose, and shape if used), and $\theta_{*}$ be the ground truth. We apply an $l_{1}$ penalty:
  (19) $$L_{\mathrm{param}}={∥\theta -\theta_{*}∥}_{1}.$$
**Object Loss.** We supervise object shape (vertex) and rigid pose:
  (20) $$L_{\mathrm{object}}=\lambda_{\mathrm{objv}}L_{\mathrm{obj}\text{-}\mathrm{vertex}}+\lambda_{\mathrm{objp}}L_{\mathrm{obj}\text{-}\mathrm{pose}}.$$
**Object vertex loss.** Let $V_{o}\in R^{N_{o}\times 3}$ be the predicted object vertices (in the canonical object frame or a chosen frame), and $V_{o,*}$ be the ground truth vertices aligned in the same frame. We use
  (21) $$L_{\mathrm{obj}\text{-}\mathrm{vertex}}=\frac{1}{N_{o}}{∥V_{o}-V_{o,*}∥}_{1}.$$
**Object pose loss.** Let (R,t) and $\left(R_{*},t_{*}\right)$ be the predicted and GT object rotation and translation. We use a translation $l_{1}$ loss and a rotation geodesic loss on SO(3):
  (22) $$L_{\mathrm{obj}\text{-}\mathrm{pose}}={∥t-t_{*}∥}_{1}+\beta_{R}\;{∥\log\;\left(R_{*}^{⊤}R\right)∥}_{1},$$
  where log(·) maps a rotation matrix to its Lie algebra (axis–angle vector).
**Hand Bounding Box Loss.** Following common practice for whole-body mesh recovery, we supervise 2D hand bounding boxes. Let $b\in R^{4}$ and $b_{*}\in R^{4}$ denote the predicted and GT hand boxes (e.g., (x,y,w,h)):
  (23) $$L_{\mathrm{hbox}}={∥b-b_{*}∥}_{1}.$$

### 3.4. Implementation Details

The model is implemented using PyTorch 1.11.0 on a single NVIDIA RTX 4090 GPU with 24 GB memory. The model is optimized using Muon [41] optimizer with learning rate 1 ×$10^{-3}$. Following previous works [12], we train the model for 50 epochs on all datasets with a batch size of 16.

## 4. Experiments

In this section, we conduct comprehensive experiments to evaluate the effectiveness of HOIMamba. We perform quantitative and qualitative analyses on two standard benchmarks and compare against existing state-of-the-art methods. Furthermore, we provide extensive ablation studies to validate the design motivations of our core components.

### 4.1. Datasets

We evaluate our method on two widely-used benchmarks for monocular image-based human–object interaction (HOI) reconstruction:

**BEHAVE Dataset** [17]: This is an indoor HOI dataset capturing interactions between 7 subjects and 20 common objects using a multi-view camera system. It provides accurate 3D ground-truth meshes for both humans (SMPL) and objects. We follow the data split defined by CHORE [11] and CONTHO [12] for a fair comparison. BEHAVE contains a rich variety of daily interactions (e.g., sitting, lifting, carrying), serving as a primary benchmark for evaluating HOI reconstruction accuracy and contact plausibility.

**InterCap Dataset** [18]: This is another large-scale indoor HOI dataset, containing interactions between 10 subjects and 10 different objects. Compared to BEHAVE, objects in InterCap possess more complex topological structures and the interaction poses are more challenging, thus better testing the model’s generalization to diversity and complexity. We adopt the standard split from prior works [12] for our experiments.

### 4.2. Evaluation Metrics

Following previous works [12], we evaluate both geometric reconstruction quality and interaction consistency using four metrics: (i) Chamfer distance on the human and object meshes, (ii) precision and recall of contact induced by reconstructed geometry.

**Mesh reconstruction accuracy.** We report Chamfer distance for the reconstructed human and object meshes, denoted as ${\mathrm{CD}}_{human}$ and ${\mathrm{CD}}_{object}$ (in centimeters). To remove global similarity ambiguities, we align the predicted and GT human–object pair via Procrustes analysis performed on the *joint* set of vertices (human and object together). After alignment, we compute Chamfer distance to the GT separately for the human mesh and the object mesh.

**Contact consistency of reconstructed geometry.** Beyond explicit contact prediction, we also quantify whether the reconstructed meshes imply plausible contact. We derive a contact label on the reconstructed human mesh by marking a human vertex as positive if its distance to the reconstructed object surface is below a fixed threshold (5 cm in all experiments). While a 5 cm threshold is standard for holistic HOI evaluation, it may be relatively coarse for fine-grained hand–object contact. We then compute precision and recall between this reconstructed contact map and the GT contact annotation, denoted as ${\mathrm{Contact}}_{p}^{rec}$ and ${\mathrm{Contact}}_{r}^{rec}$, respectively.

### 4.3. Quantitative Results and Comparisons

Table 1 reports quantitative comparisons on BEHAVE and InterCap. HOIMamba achieves consistent improvements over representative prior methods across both geometric accuracy (Chamfer distance) and contact consistency. On BEHAVE, HOIMamba reduces ${\mathrm{CD}}_{human}$ from 4.99 cm to 4.56 cm and ${\mathrm{CD}}_{object}$ from 8.42 cm to 7.91 cm compared with the strongest baseline CONTHO. Meanwhile, contact precision increases from 0.628 to 0.658 and contact recall improves from 0.496 to 0.563, indicating that the reconstructed geometry not only matches the ground truth more closely but also implies more correct contact regions.

On InterCap, the same trend holds. HOIMamba improves ${\mathrm{CD}}_{human}$ from 5.96 cm to 5.52 cm and ${\mathrm{CD}}_{object}$ from 9.50 cm to 8.84 cm, while boosting contact precision from 0.661 to 0.690 and contact recall from 0.432 to 0.500. Notably, the recall gains on both benchmarks suggest that HOIMamba is better at recovering interaction-critical regions (e.g., hands and forearms in grasping/carrying) that are often missing or misaligned under occlusion. These results support our theory that structured state evolution provides a more suitable inductive bias than isotropic all-to-all mixing for capturing asymmetric and localized physical interactions. These improvements are especially relevant for contact-driven scenarios, where small errors around hands or support regions can change the interaction interpretation.

**Runtime Analysis.** We also compare the runtime of HOIMamba with the attention-based baseline, i.e., CONTHO [12]. We use the same hardware configuration as in Table 1 and measure the average time per image and memory usage in seconds. CONTHO takes 0.1 s per image with a peak memory usage of 928 MB, while HOIMamba takes 0.07 s per image with a peak memory usage of 884 MB. This suggests that HOIMamba is able to achieve comparable runtime and memory usage while achieving higher accuracy.

### 4.4. Qualitative Results

**Figure 4:**
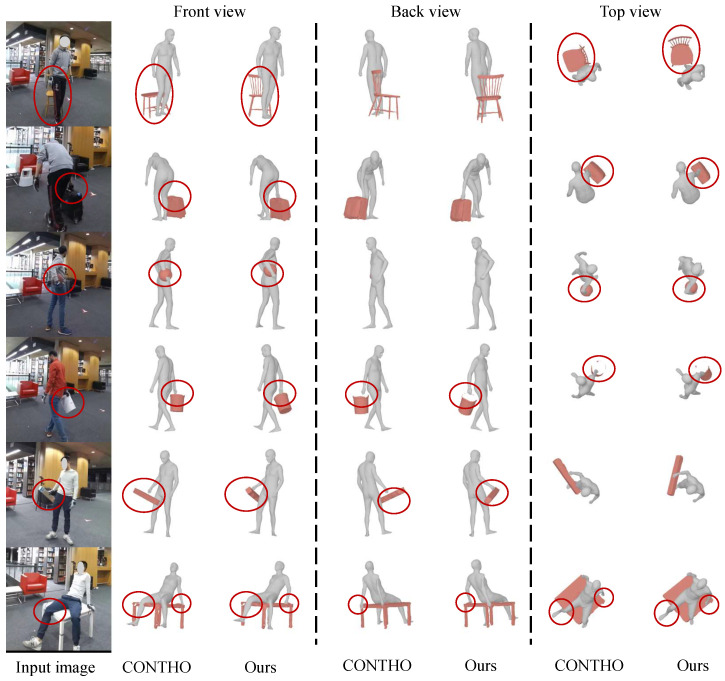
Qualitative comparison of HOIMamba against prior methods on BEHAVE dataset. HOIMamba produces more accurate human and object interaction, with improved contact localization and reduced interpenetration.

We further provide qualitative comparisons on BEHAVE in Figure 4. Compared with prior methods, HOIMamba reconstructs more physically plausible interactions with (i) tighter alignment in contact-sensitive regions and (ii) reduced visible interpenetration. In challenging cases with strong mutual occlusion or small contact areas, baseline reconstructions may exhibit floating contact or mismatched hand–object alignment. In contrast, HOIMamba better preserves the relative configuration between the hand/arm and the object surface, producing interaction states that are both globally coherent and locally consistent. These observations are consistent with the quantitative gains in contact precision/recall in Table 1, suggesting that the proposed state-space interaction modeling improves not only overall mesh accuracy but also the locality and correctness of contact implied by reconstructed geometry.

### 4.5. Limitations

**Figure 5:**
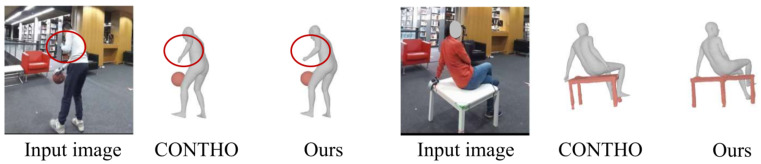
Representative failure cases of HOIMamba. In some challenging scenarios with rare or complex poses, the model may produce inaccurate human pose estimation, leading to reduced reconstruction quality.

Although HOIMamba demonstrates consistent improvements across benchmarks, some challenging cases are still difficult. Figure 5 shows some examples of our failure cases. In particular, when the interaction involves rare or complex poses that are rare in the training data distribution, the model may produce inaccurate human pose estimation. Such errors are partly due to the inherent ambiguity of monocular reconstruction and the limited coverage of long-tail distributions in existing datasets. Notably, similar difficulties are also observed in prior methods under these conditions. Additionally, we set the number of object vertices to 64, which may limit the reconstruction of fine geometric details for objects with complex topologies. Moreover, note that for objects without known templates, one would need to rely on category-level shape priors or template-free reconstruction methods, which is an interesting direction for future research.

Future works may include reconstructing stronger pose priors with denser object models, synthetic data augmentation for rare interactions, or leveraging multi-view cues to reduce ambiguity. Moreover, while our geometric losses and contact evaluation metrics provide indirect supervision, we will incorporate explicit physics-inspired losses to improve the realism of reconstructions, especially in tight hand–object contact regions.

## 5. Ablation Study

To thoroughly analyze the effectiveness of each design choice in HOIMamba, we conduct a series of controlled ablation experiments on the BEHAVE validation set unless otherwise specified. All ablated variants are trained under identical settings and evaluated using the same protocols. Our analysis focuses on four key aspects: (i) the necessity of state-space modeling over attention-based alternatives, (ii) the role of multi-scale state inference, (iii) the factorization of spatial and channel-wise interaction reasoning, and (iv) the importance of explicit bidirectional human–object state propagation.

### 5.1. Ablation Settings and Variant Definitions

To avoid ambiguity, we clarify each variant in Table 2. *Attention-based* denotes a baseline that replaces the state-space blocks with standard self-attention while keeping the same tokenization strategy and output heads. *Single-scale SSM* replaces MSSM with a single SSM branch (i.e., without multi-scale grouping). *h→o* and *o→h* denote unidirectional interaction propagation, where state updates are performed only from human tokens to object tokens or vice versa. *w/o Channel* removes the channel-state pathway in SCSSM and retains only token-wise (spatial) state evolution, while *w/o Spatial* removes the spatial pathway and keeps only channel-wise state evolution. *Single-path SSM* denotes a simplified design that uses only one pathway (token-wise or channel-wise) without spatial-channel factorization and gated fusion. The full model (*HOIMamba (SSM)*) uses MSSM + SCSSM with explicit bidirectional propagation.

### 5.2. Impact of State-Space Modeling

We first examine whether replacing Transformer-style self-attention with state-space modeling provides tangible benefits beyond computational efficiency. To this end, we construct an attention-based baseline [12] by replacing all MSSM blocks with standard multi-head self-attention layers of comparable depth and parameter count. Both variants employ identical tokenization strategies and output heads to ensure a fair comparison.

As shown in Table 2, replacing attention with state-space modeling consistently improves both reconstruction accuracy and contact consistency. In particular, contact recall increases from 0.496 to 0.563 when moving from the attention-based baseline to the full HOIMamba model, indicating better recovery of interaction regions that are easily missed under occlusion. These gains suggest that the advantage of HOIMamba stems not only from linear-time computation, but also from a more suitable inductive bias for structured interaction reasoning, whereas attention-based mixing may remain globally coherent yet locally imprecise in contact-sensitive regions.

### 5.3. Effect of Multi-Scale State-Space Modeling

Human–object interactions contain cues at multiple spatial extents. Hand–object contact provides fine-grained local signals. Body–object coordination provides global signals. We study this property with a controlled comparison. We compare the proposed multi-scale state-space module (MSSM) with a single-scale SSM variant. The single-scale variant uses a fixed resolution.

Table 2 reports the results. Multi-scale state inference improves performance over the single-scale variant. Specifically, MSSM reduces ${\mathrm{CD}}_{human}$ from 4.83 to 4.56, and reduces ${\mathrm{CD}}_{object}$ from 8.26 to 7.91. Moreover, MSSM improves contact recall from 0.502 to 0.563, which suggests that hierarchical state propagation captures this aggregation better than fixed-scale updates. These gains support our theory that multi-granularity aggregation is beneficial for interaction reasoning.

### 5.4. Spatial-Channel Factorization Analysis

In this section, we study the effectiveness of spatial-channel grouped state-space modeling (SCSSM). To this end, we evaluate two degraded variants for ablation. The first variant is *w/o Channel*. This variant removes channel-wise state evolution and keeps only token-wise (spatial) state evolution. The second variant is *w/o Spatial*. This variant removes token-wise (spatial) state evolution and keeps only channel-wise state evolution.

Table 2 shows that each removal hurts performance. The *w/o Spatial* variant causes a large degradation. In this case, ${\mathrm{CD}}_{human}$ rises to 7.96. Contact metrics also drop substantially in this setting. This result highlights the role of token-wise state evolution, which demonstrates that the spatial pathway is necessary for geometric localization and accurate contact modeling. The *w/o Channel* variant shows a smaller but consistent drop. This trend suggests a complementary role for channel-wise state evolution. The channel pathway improves semantic and functional consistency of interaction features, which can improve contact recall and object alignment. Overall, these results validate the spatial-channel factorization design. The factorization helps the model separate where interaction occurs from how it is semantically realized.

### 5.5. Bidirectional Interaction Reasoning

Finally, we evaluate the importance of explicit bidirectional human–object state propagation. We compare the proposed bidirectional scheme against two unidirectional alternatives: human-to-object only and object-to-human only propagation.

As shown in Table 2, bidirectional reasoning yields the best overall performance. Interestingly, the unidirectional variants already perform competitively, reflecting the inherently asymmetric nature of physical interactions in which one entity may provide stronger constraints depending on the interaction type. Nevertheless, incorporating the reverse-direction feedback further refines the interaction state and improves contact consistency, leading to the best geometric and contact-aware metrics in the full model.

### 5.6. Discussion

Overall, the ablation results demonstrate that each component of HOIMamba contributes meaningfully and synergistically to performance. The observed gains cannot be attributed solely to increased model capacity, but rather to principled inductive biases tailored for interaction-centric reasoning. These findings support our central claim that monocular HOI reconstruction benefits from structured, directional, and disentangled state inference, rather than symmetric feature correlation.

## 6. Conclusions

In this paper, we presented HOIMamba, a bidirectional state-space modeling framework for monocular 3D human–object interaction reconstruction. By reformulating HOI reconstruction as structured interaction state inference, HOIMamba replaces isotropic all-to-all mixing with controlled state evolution that better matches the asymmetric and locality-sensitive nature of physical interactions. We introduced (i) a multi-scale state-space module (MSSM) to capture interaction cues across granularities and (ii) a spatial-channel grouped SSM (SCSSM) block to decouple geometric/contact dependencies from semantic/functional correlations, followed by gated fusion. Extensive experiments on BEHAVE and InterCap demonstrate that HOIMamba consistently improves both mesh reconstruction accuracy and contact consistency over representative prior methods. Ablation studies further verify that state-space modeling, multi-scale inference, spatial-channel factorization, and explicit bidirectional propagation each contribute meaningfully and synergistically to the final performance. We believe HOIMamba provides a scalable and effective alternative to attention-based HOI reconstruction and offers a promising direction for structured interaction reasoning in broader 3D vision tasks. A natural next step is to integrate the perception module into a collaborative robot pipeline to evaluate its impact on intent prediction and safe co-manipulation.

## Figures and Tables

**Table 1:** Quantitative comparison on BEHAVE and InterCap datasets. The best metrics have been highlighted using **bold** font.

**Methods**	**BEHAVE**			
	**CD_*human*_↓**	**CD_*object*_↓**	**Contact_*p*_↑**	**Contact_*r*_↑**
PHOSA [10]	12.17	26.62	0.393	0.266
CHORE [11]	5.58	10.66	0.587	0.472
CONTHO [12]	4.99	8.42	0.628	0.496
HOIMamba (Ours)	**4.56**	**7.91**	**0.658**	**0.563**
**Methods**	**InterCap**			
	**CD_*human*_↓**	**CD_*object*_↓**	**Contact_*p*_↑**	**Contact_*r*_↑**
PHOSA [10]	11.20	20.57	0.228	0.159
CHORE [11]	7.01	12.81	0.339	0.253
CONTHO [12]	5.96	9.50	0.661	0.432
HOIMamba (Ours)	**5.52**	**8.84**	**0.690**	**0.500**

**Table 2:** Ablation study on the BEHAVE dataset. The best metrics have been highlighted using **bold** font.

Method	CD_*human*_↓	CD_*object*_↓	Contact_*p*_↑	Contact_*r*_↑
Attention-based [12]	4.99	8.42	0.628	0.496
Single-scale SSM	4.83	8.26	0.631	0.502
w/o Channel	4.65	8.32	0.640	0.558
w/o Spatial	7.96	11.10	0.476	0.421
h→o	4.62	8.37	0.642	0.553
o→h	4.63	8.03	**0.658**	0.549
HOIMamba (SSM)	**4.56**	**7.91**	**0.658**	**0.563**

## Data Availability

The data analyzed in this study were obtained from publicly available datasets. The specific datasets and access information are as follows: **BEHAVE** (available at https://virtualhumans.mpi-inf.mpg.de/behave/, accessed on 8 February 2026) and **InterCap** (available at https://intercap.is.tue.mpg.de/, accessed on 8 February 2026). No new raw data were created in this study.

## Abbreviations

The following abbreviations are used in this manuscript:

HOI	Human–Object Interaction
SSM	Structured State-Space Model
MSSM	Multi-Scale Structured State-Space Model
SCSSM	Spatial-Channel Grouped Structured State-Space Model
SMPL	Skinned Multi-Person Linear model
